# Impact of Mediterranean Diet on Disease Activity and Gut Microbiota Composition of Rheumatoid Arthritis Patients

**DOI:** 10.3390/microorganisms8121989

**Published:** 2020-12-14

**Authors:** Andrea Picchianti Diamanti, Concetta Panebianco, Gerardo Salerno, Roberta Di Rosa, Simonetta Salemi, Maria Laura Sorgi, Giorgia Meneguzzi, Maria Benedetta Mariani, Alessandra Rai, Dalila Iacono, Giorgio Sesti, Valerio Pazienza, Bruno Laganà

**Affiliations:** 1 Department of Clinical and Molecular Medicine, Sant’Andrea University Hospital, Sapienza University of Rome, 00185 Rome, Italy; gerardo.salerno@uniroma1.it (G.S.); roberta.dirosa@uniroma1.it (R.D.R.); simonetta.salemi@ospedalesantandrea.it (S.S.); marialaura.sorgi@uniroma1.it (M.L.S.); giorgia.meneguzzi@uniroma1.it (G.M.); mariabenedetta.mariani@uniroma1.it (M.B.M.); rai.1635398@studenti.uniroma1.it (A.R.); dalilaiacono@gmail.com (D.I.); giorgio.sesti@uniroma1.it (G.S.); bruno.lagana@uniroma1.it (B.L.); 2 Division of Gastroenterology, Fondazione IRCCS Casa Sollievo della Sofferenza Hospital, 71013 San Giovanni Rotondo, Italy; panebianco.c@gmail.com

**Keywords:** gut microbiota, rheumatoid arthritis, Mediterranean diet, disease activity

## Abstract

Rheumatoid arthritis (RA) is an autoimmune disorder in which gut and oral microbiota play a crucial role. Diet is a modifiable factor that can influence both microbiota composition and arthritis outcome; previous studies have suggested associations between dietary habits and RA, with contrasting results. We investigate the protective effect of the Mediterranean diet (MD) on disease activity and the gut microbiota profile in RA patients. Sixty consecutive RA patients were enrolled upon filling a validated 14-item questionnaire for the assessment of adherence to the Mediterranean diet (Prevention with Mediterranean Diet-PREDIMED). Then, 16S analysis was employed to explore the gut microbiota within the two cohorts of patients. Patients with high adherence to MD (20) had a significantly lower C-reactive protein (*p* < 0.037) and disease activity (*p* < 0.034) than the 40 patients with low/moderate adherence to MD. An inverse association between MD and disease activity was confirmed by multivariate analysis after adjustments for all the different demographic, clinical and serologic variables. A healthier gut microbiota composition was observed in the high adherence group, with a significant decrease in Lactobacillaceae and an almost complete absence of *Prevotella copri* with respect to the low/moderate adherence group. In conclusion, our findings support the protective role of MD on disease activity and microbiota composition in RA patients, and suggest the feasibility of shifting the habitual diet to modulate the gut microbiota and promote the benefits associated with MD.

## 1. Introduction

Rheumatoid arthritis (RA) is a chronic autoimmune disease of unknown etiology, in which gut and oral microbiota play a crucial role in modulating innate and acquired immune responses, and thus interfering with the fragile balance between inflammation and tolerance. Translocation of intestinal microbes, enabled by the increased permeability of the intestinal epithelial cell layer, or enhanced exposure to microbial products, have been suggested as possible links connecting gut dysbiosis and joint pathology [1]. In the last two decades, different abnormalities in the gut and oral microbiota composition of RA patients have been described, among which a decreased gut microbial diversity with a lower representation of common commensals such as *Bifidobacteria and Bacteroides* species, as well as an increase in *Prevotella copri spp, Escherichia coli*, *Mycoplasma fermentans*, *Proteus mirabilis, Colinsella, Faecalibacterium* and *Lactobacillus* communities [2,3,4,5,6,7,8]. We have also recently demonstrated that RA patients present specific gut microbiota abnormalities, some of which were correlated with disease activity; moreover, the treatment with the anti-TNFα biologic agent etanercept was able to partially restore a healthy microbiota composition [9]. Diet is a modifiable environmental factor that can influence both microbiota composition and arthritis outcome. A possible cause–effect relation between diet and arthritis is hard to demonstrate because genetics and living conditions cannot be ruled out as relevant biases. However, the impact of diet on the gut microbiota is suggested by the differences in its composition/variety between geographically and life-style distant populations [10]. It is known, indeed, that a diet rich in animal proteins, simple sugars, and saturated fats, typical of western countries, is characterized by a reduction in the variety of microbiomes and is associated with the *Bacteroides* enterotype, whereas *Prevotella* is the most prevalent in a diet habit rich in fruits and vegetables [11]. In contrast to the western diet, the Mediterranean diet is considered a healthy eating pattern, associated with reduced risk for psoriasis, metabolic, cardiovascular, and neoplastic diseases [12,13,14,15]. One of the most accredited hypotheses of this association is that the high content of different beneficial compounds, such as antioxidants and polyphenols, largely present in Mediterranean foods, such as plant foods, fruits, and red wine, have anti-inflammatory properties [16]. Dietary intervention can also significantly modify gut microbiota structure and richness. Foods rich in fibers, such as those present in the Mediterranean diet, indeed, are degraded by *Firmicutes* and *Bacteroidetes* into short-chain fatty acids (SCFA), such as butyrate, [17,18], which can result in a protective effect on the intestinal barrier by reducing its permeability [19]. A number of studies have also suggested associations between dietary habits, and RA development and outcome; however, the results are still scarce and contrasting [20,21,22,23,24,25,26,27,28,29]. In this study, we found a protective role of the Mediterranean diet on the disease activity of RA patients supported by a healthy impact on microbiota composition.

## 2. Materials and Methods

### 2.1. Study Population

Sixty consecutive RA patients, diagnosed according to the European League Against Rheumatism (EULAR)/American College of Rheumatology (ACR) classification criteria [29], were followed at the outpatient Division of Immunology and Rheumatology, S. Andrea Hospital, Sapienza University of Rome. All subjects gave their written informed consent before they participated in the study and the study was conducted in accordance with the Declaration of Helsinki. All patients were receiving current therapy for at least three months and they were not taking steroids, non-steroidal anti-inflammatory drugs (NSAIDs) and proton pumps inhibitors during the 2 weeks before the exams. Any patients on antibiotics, consuming probiotics, progestins or having a known history of inflammatory bowel disease and endocrinopathies were excluded.

Demographic data, dietary and smoking habits, comorbidities, therapeutic regimen, disease activity score on 28 joints (DAS28), erythrocyte sedimentation rate (ESR), C-reactive protein (CRP), rheumatoid factor (RF), anti-cyclic citrullinated peptides antibodies (ACPA) were also registered.

In each subject, weight and height were used to calculate the body mass index (BMI) (weight (kg) divided by height squared (m^2^), kg/m^2^). The degree of obesity was established according to a scale based on BMI cut-off points: 18.5 < 25 kg/m^2^ normal weight, 25 ≤ 30 kg/m^2^ overweight, >30 kg/m^2^ obesity, respectively. A validated 14-item questionnaire for the assessment of adherence to the Mediterranean diet Prevention with Mediterranean Diet-PREDIMED) [30] was recorded for all the enrolled subjects during a face-to-face interview between the patient and a rheumatologist. Briefly, each item was assigned score 1 and 0; PREDIMED score was calculated as follows: 0–5, low adherence; 6–9, moderate adherence; ≥10, high adherence.

### 2.2. Sample Collection and DNA Extraction

First, 300 µL of fresh stool samples collected in a tube filled with a DNA stabilization buffer (Canvax Biotech, Cordoba, Spain) was processed for microbial genomic DNA extraction using the QIAamp DNA Stool Mini Kit (Qiagen, Milan, Italy) according to the manufacturer’s protocol. DNA concentration and purity were assessed by a NanoDrop spectrophotometer (Thermo Scientific, Waltham, MA, USA).

### 2.3. Next-Generation Sequencing of Bacterial 16S rRNA Gene 

As routinely performed in our laboratory, the V3–V4 hypervariable regions of the bacterial gene encoding 16S ribosomal RNA were amplified by polymerase chain reaction (PCR) using barcoded universal primers [31]. The PCR products were purified, quantified and equimolar ratios of amplicons from individual samples were pooled before sequencing on the Illumina MiSeq platform using high throughput screening. 

### 2.4. Bioinformatic Analysis

Demultiplexed sequence data were analyzed using 16S Metagenomics GAIA 2.0 software, which performs quality control of the reads/pairs (i.e., trimming, clipping and adapter removal steps) through FastQC and BBDuk as previously reported [32]. The read pairs were mapped with BWA-MEM against the custom databases (based on NCBI) for taxonomy assignment. Alpha diversity index (Shannon) was also computed. As for microbiota composition, the differential analysis was performed by the DESeq2 analysis. Results were considered significant when *p* < 0.05 and FDR < 0.05.

### 2.5. Statistical Analysis

Continuous data were described by median (25–75th percentil), while the categorical variables were described as percentages (%). The D’Agostino–Pearson test was used to test the normality of data. Fisher’s exact test was used for analysis of the contingency table, while the Mann–Whitney test was used to compare ranks. Using DAS28 as a dependent variable, a multiple linear regression analysis model was set up considering for multivariable analysis every variable with *p* < 0.05 in univariate analysis. Correlation analyses were performed using Spearman rank correlation. Statistical analysis was performed using “Frisbee Sailing” R version 3.0.2 (25 September 2013, copyright 2013), the R Foundation for Statistical Computing. Student’s t-test was performed to compare diversity metrics. Results were considered significant when *p* < 0.05.

## 3. Results

Main demographic, anthropometric, and clinical characteristics of the RA patients are shown in Table 1.

### 3.1. Adherence to Mediterranean Diet and Clinical Characteristics of RA Patients

Considering that only three patients had a low adherence score to the Mediterranean diet, we decided to divide the patients into two groups: low/moderate adherence (40 patients) and high adherence (20 patients).

As reported in Table 2, patients with high adherence to the Mediterranean diet had a significantly lower disease activity (*p* < 0.034) and CRP (*p* < 0.037); on the other hand, they presented a significantly higher disease duration (*p* < 0.033) and a prevalence of the female gender (*p*< 0.023). 

By using DAS28 as a dependent variable, we looked at possible associations between the level of adherence to the Mediterranean diet and the main demographic, anthropometric and clinical parameters (i.e., sex, age, BMI, disease duration, DAS28, RF, ACPA, ESR and CRP) of the RA patients. 

As reported in Table 3, the univariate analysis demonstrated an inverse correlation with the class of adherence to the Mediterranean diet (*p* < 0.047), smoking habit (*p* < 0.049) and BMI (*p* < 0.042) and a direct correlation with the ESR (*p* < 0.016). Then, a multiple linear regression analysis model was set up considering for multivariable analysis every variable with *p* < 0.05 in univariate analysis. This approach confirmed the inverse correlation between the DAS28 and the Mediterranean diet (*p* < 0.042) and the direct correlation with the ESR (*p* < 0.017). 

### 3.2. Microbiota Profile in RA Patients with Low/Moderate and High Adherence to Mediterranean Diet

We next sought to evaluate any difference in fecal microbiota composition of RA patients based on whether they were adherent or not to the Mediterranean diet. On average, 164,475 ± 70,544 quality-filtered sequence pairs were generated per sample. 

**Figure 1:**
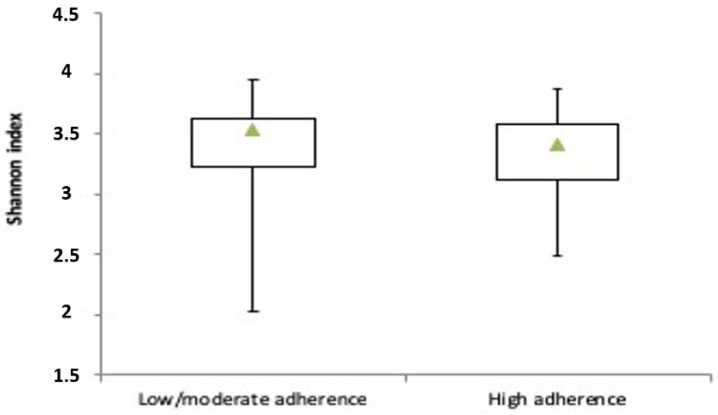
Alpha-diversity metrics in low/moderate adherence and high adherence to the Mediterranean diet groups. Box plots of species-level Shannon diversity index.

The calculation of diversity metrics at species level revealed that the Mediterranean diet had no significant impact on alpha-diversity as expressed by the Shannon index (Figure 1).

**Figure 2:**
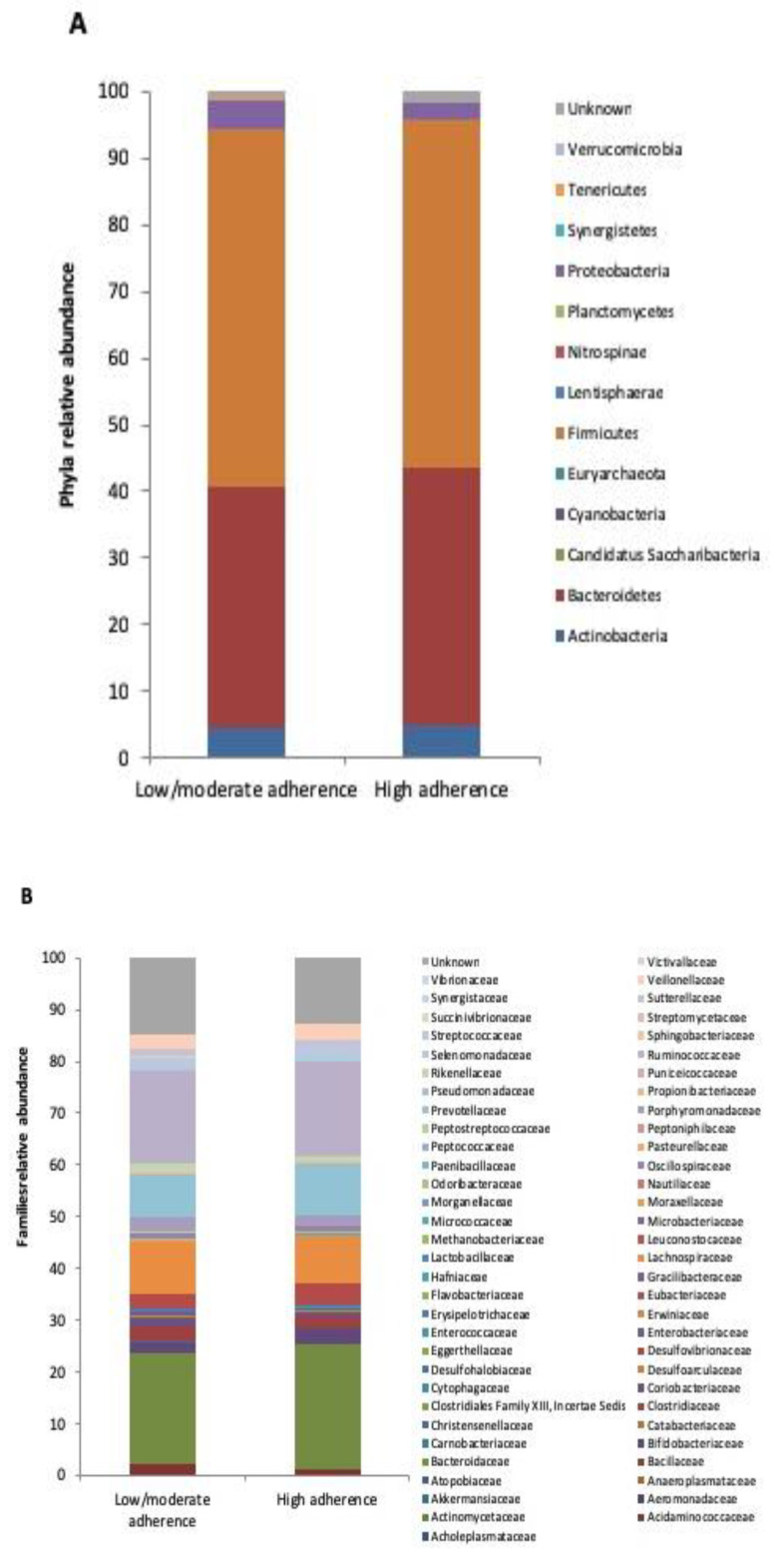
Microbiota composition in low/moderate adherence and high adherence to the Mediterranean diet groups. Mean relative abundance (%) at phyla (**A**) and families (**B**) levels.

Concerning the microbial communities’ composition, the two diet groups exhibited a remarkable number of significant changes in each of the taxonomic levels analyzed. At phylum level (Figure 2A), Cyanobacteria were over-represented in high adherence compared to low/moderate adherence patients (0.010% vs. 0%), whereas the opposite was observed for Euryarchaeota (0.011% vs. 0.061%), Nitrospinae (0 % vs. 0.072%), Planctomycetes (0% vs. 0.011%), Tenericutes (0.044% vs. 0.355%) and Verrucomicrobia (0.024% vs. 0.068%).

Paenibacillaceae was the only family enriched in the high adherence versus low/moderate adherence group (0.012% vs. 0%), while Acholeplasmataceae (0% vs. 0.051%), Anaeroplasmataceae (0% vs. 0.019%), Atopobiaceae (0.011% vs. 0.075%), Carnobacteriaceae (0% vs. 0.014%), Clostridiaceae (1.905% vs. 2.610%), Clostridiales Family XIII, Incertae Sedis (0% vs. 0.026%), Cytophagaceae (0% vs. 0.015%), Desulphohalobiaceae (0% vs. 0.013%), Flavobacteriaceae (0% vs. 0.014%), Gracilibacteraceae (0% vs. 0.015%), Lactobacillaceae (0.159% vs. 0.229%), Leuconostocaceae (0% vs. 0.011%), Methanobacteriaceae (0.011% vs. 0.060%), Microbacteriaceae (0% vs. 0.011%), Nautiliaceae (0% vs. 0.031%), Peptostreptococcaceae (0.049% vs. 0.096%), Puniceicoccaceae (0% vs. 0.038%), Sphingobacteriaceae (0% vs. 0.019%) and Synergistaceae (0% vs. 0.021%) were all depleted (Figure 2B).

**Figure 3:**
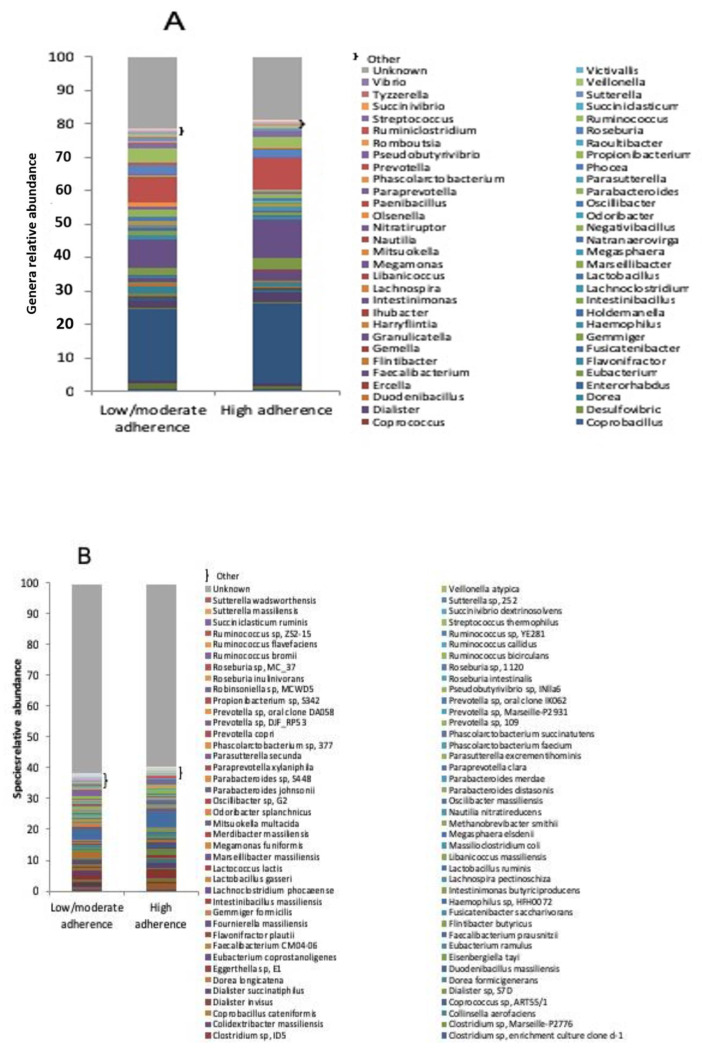
Microbiota composition in low/moderate adherence and high adherence to the Mediterranean diet groups. Mean relative abundance (%) of bacterial genera (**A**) and species (**B**).

Within the genera (Figure 3A), the high adherence group showed a greater abundance of *Candidatus Saccharimonas* (0.012% vs. 0%), *Coprobacillus* (0.023% vs. 0%), *Intestinibacillus* (0.074% vs. 0%), *Nitratiruptor* (0.011% vs. 0%) and *Paenibacillus* (0.012% vs. 0%), while, on the contrary, a lower abundance of *Arsenophonus* (0% vs. 0.013%), *Bariatricus* (0% vs. 0.015%), *Bittarella* (0% vs. 0.011%), *Blautia* (0.685% vs. 0.910%), *Candidatus Phytoplasma* (0% vs. 0.017%), *Candidatus Soleaferrea* (0.012% vs. 0.049%), *Catabacter* (0% vs. 0.019%), *Clostridioides* (0% vs. 0.010%), *Desulfovibrio* (0.035% vs. 0.103%), *Dorea* (0.248% vs. 0.467%), *Duodenibacillus* (0.058% vs. 0.085%), *Enterohabdus* (0.031% vs. 0.055%), *Flintibacter* (0.017% vs. 0.039%), *Gemella* (0% vs. 0.010%), *Granulicatella* (0% vs. 0.013%), *Harryflintia* (0% vs. 0.018%), *Ihubacter* (0% vs. 0.017%), *Lactobacillus* (0.157% vs. 0.205%), *Libanicoccus* (0% vs. 0.043%), *Natranaerovirga* (0.028% vs. 0.033%), *Nautilia* (0% vs. 0.029%), *Olsenella* (0% vs. 0.026%), *Phocea* (0% vs. 0.018%), *Propionibacterium* (0.160% vs. 0.473%), *Provencibacterium* (0% vs. 0.026%), *Raoultibacter* (0% vs. 0.017%), *Romboutsia* (0.011% vs. 0.036%), *Selenomonas* (0% vs. 0.010%), *Vibrio* (0% vs. 0.010%) and *Victivallis* (0% vs. 0.018%).

Finally, at species level (Figure 3B), patients with high adherence to the Mediterranean diet had an over-representation, compared to low/moderate adherence group, in: *Adlercreutzia equolifaciens* (0.014% vs. 0%), *Anaeromassilibacillus senegalensis* (0.011% vs. 0%), *Bacteroides coprophilus* (0.441% vs. 0.045%), *Bacteroides fragilis* (1.216% vs. 0.081%), *Bacteroides* *thetaiotaomicron* (0.743% vs. 0.161%), *Clostridium lavalense* (0.014% vs. 0%), *Clostridium scindens* (0.013% vs. 0%), *Clostridium sp. enrichment culture clone d-1* (0.017% vs. 0%), *Coprobacillus cateniformis* (0.022% vs. 0%), *Dialister sp. S7D* (0.628% vs. 0.024%), *Fournierella massiliensis* (0.047% vs. 0%), *Intestinibacillus massiliensis* (0.068% vs. 0%), *Lachnoclostridium phocaeense* (0.015% vs. 0%), *Lactococcus lactis* (0.040% vs. 0%) and *Massilioclostridium coli* (0.016% vs. 0%). 

Vice versa, the following species were all under-represented in high adherence with respect to low/moderate adherence subjects: *Alistipes ihumii* (0% vs. 0.038%), *Alistipes massiliensis* (0% vs. 0.033%), *Alistipes sp. cv1* (0% vs. 0.028%), *Alistipes sp. NML05A004* (0% vs. 0.023%), *Arsenophonus endosymbiont of Bemisia tabaci* (0% vs. 0.011%), *Bacteroides barnesiae* (0% vs. 0.134%), *Bacteroides clarus* (0% vs. 0.013%), *Bacteroides oleiciplenus* (0% vs. 0.013%), *Bacteroides sp. ANH 2438* (0.143% vs. 0.164%), *Bacteroides stercoris* (0.013% vs. 0.047%), *Bacteroides uniformis* (0.078% vs. 0.171%), *Butyricimonas virosa* (0.011% vs. 0.058%), *Citrobacter freundii* (0% vs. 0.045%), *Clostridium sp. 826* (0% vs. 0.190%), *Clostridium sp. AT4* (0.011% vs. 0.281%), *Clostridium sp. BPY5* (0% vs. 0.011%), *Clostridium sp. Culture Jar-8* (0% vs. 0.017%), *Clostridium sp. enrichment culture clone 06-1235251-89* (0% vs. 0.019%), *Clostridium sp. ID5* (0% vs. 0.013%), *Clostridium sp. Marseille-P2776* (0% vs. 0.013%), *Colidextribacter massiliensis* (0% vs. 0.014%), *Dorea formicigenerans* (0.028% vs. 0.059%), *Duodenobacillus massiliensis* (0.057% vs. 0.083%), *Eggerthella sp. E1* (0% vs. 0.028%), *Eisenbergiella tayi* (0% vs. 0.022%), *Flintibacter butyricus* (0.012% vs. 0.026%), *Haemophilus sp. HFH0072* (0% vs. 0.012%), *Intestinimonas butyriciproducens* (0% vs. 0.018%), *Lactobacillus gasseri* (0% vs. 0.063%), *Libanococcus massiliensis* (0% vs. 0.038%), *Merdibacter massiliensis* (0% vs. 0.014%), *Methanobrevibacter smithii* (0% vs. 0.046%), *Nautilia nitratireducens* (0% vs. 0.015%), *Parabacteroides johnsonii* (0.039% vs. 0.126%), *Parabacteroides merdae* (0% vs. 0.114%), *Paraprevotella xylaniphila* (0.041% vs. 0.148%), *Parasutterella secunda* (0.039% vs. 0.126%), *Phascolarctobacterium faecium* (0% vs. 0.489%), *Phascolarctobacterium sp. 377* (0% vs. 0.303%), *Prevotella copri* (0% vs. 0.071%), *Prevotella sp. DJF_RP53* (0% vs. 0.296%), *Prevotella sp. Marseille-P2931* (0% vs. 0.233%), *Prevotella sp. oral clone DA058* (0% vs. 0.043%), *Propionibacterium sp. S342* (0.154% vs. 0.457%), *Robinsoniella sp. MCWD5* (0% vs. 0.011%), *Roseburia sp. MC_37* (0.058% vs. 0.155%), *Ruminococcus flavefaciens* (0% vs. 0.025%), *Ruminococcus sp. YE281* (0% vs. 0.011%), *Ruminococcus sp. ZS2-15* (0% vs. 0.020%), *Sutterella sp. 252* (0% vs. 0.076%) and *Veillonella atypica* (0% vs. 0.015%).

With the aim of establishing any association between microbial taxa and the adherence to the Mediterranean diet, correlation analyses were performed.

**Figure 4:**
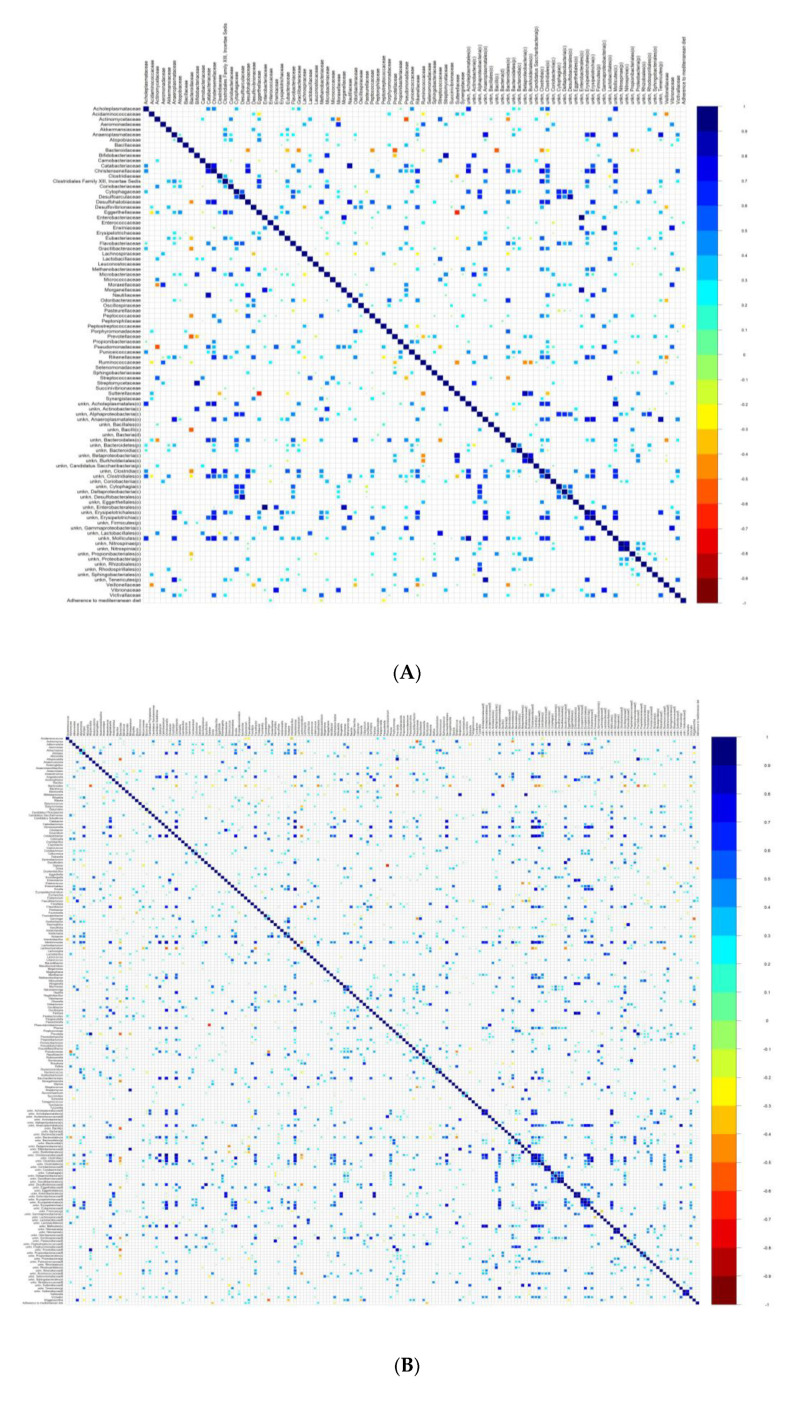

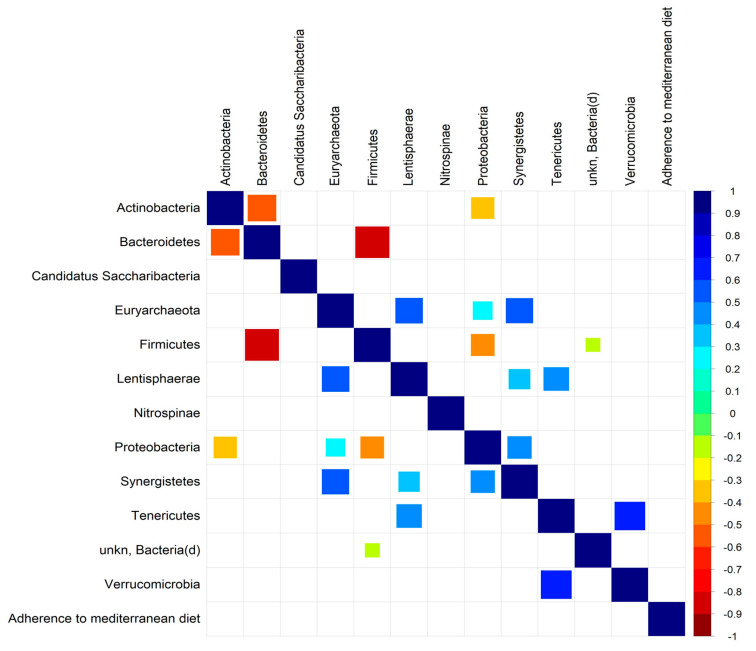
Association of gut microbiota profile with clinical pathological features in RA patients at the family (**A**), genus (**B**), and phylum (**C**) levels.

The Spearman’s correlation analysis revealed a direct association between high adherence to the Mediterranean diet and Enterococcaceae, whereas an inverse correlation was found with Methanobacteriaceae and Peptostreptococcaceae. At genus level, a direct association was found with *Coprobacillus*, *Eubacterium*, *Faecalibacterium*, *Marseillibacter* and *Tetragenococcus* and an inverse correlation with *Dorea*, *Methanobrevibacter*, *Romboutsia* (Figure 4).

## 4. Discussion

In this study, we tried to assess potential inverse associations between the degree of adherence to the Mediterranean diet and disease activity and identify possible correlations with the gut microbiota characteristics in RA patients. As hypothesized, the comparison between patients with high and low/moderate adherence to the Mediterranean diet revealed that the first group had significantly lower disease activity expressed by the DAS28, and a lower level of systemic inflammation expressed by CRP. Furthermore, the univariate analysis demonstrated an inverse correlation between the DAS28 and the class of adherence to the Mediterranean diet, a result confirmed by the multivariate analysis after adjustments for all the different demographic, anthropometric, clinical, and serologic variables. The protective role of the Mediterranean diet on disease activity of RA patients was supported by a healthy impact on microbiota composition.

In the last two decades, the advances in sequencing technologies have led to a growing awareness of the role played by the microbiota in the pathogenesis and outcome of autoimmune arthritis. It has also been suggested that the gut microbiota can be significantly modulated by different environmental factors such as diet, smoke, and therapy [1,10]. On the other hand, it has been reported, with contrasting results, that diet regimen can influence arthritis onset and outcome [20,21,22,23,24,25,26,27,28,29]. Unlike the western diet, the Mediterranean diet is composed of a variety of foods and has been reported as the best-balanced and complete diet that provides antioxidant, anti-inflammatory, and prebiotic effects [12,13]. The Mediterranean diet, indeed, is characterized by a significant amount of unrefined cereals, fruit, vegetables, legumes; a consistent intake of extra-virgin olive oil, fish; a moderate consumption of eggs, dairy products, alcohol and a low consumption of sweets and red meat [17]. Foods rich in fibers such as those assumed in the Mediterranean diet are mainly degraded by Firmicutes and Bacteroides into SCFA such as butyrate, which could have positive impact on gut dysbiosis by reducing intestinal permeability, bacterial translocation, and limit inflammation [17,19]. By now, a few studies have specifically evaluated the effect of the Mediterranean diet on RA patients, with mostly inconclusive results. Two authors have failed to prove that adherence to the Mediterranean diet has a significant protective effect on the risk of RA development [33,34], whereas a more recent large population-based case–control study has revealed that the Mediterranean diet score was inversely associated with risk of RA onset [35]. However, this effect was observed only among men and seropositive patients, a subgroup not specifically analyzed in the previous studies. There is suggestive but insufficient evidence for a positive impact of the Mediterranean diet on RA-related outcomes (i.e., pain, physical function, disease activity), as reported by a 2009 Cochrane review and recently confirmed by two systematic reviews on the role of Mediterranean diet in RA patients [36,37,38]. In particular, a RCT found that a 12 week Mediterranean diet could reduce disease activity (mainly tender joints and general health) but was unable to rescue physical function or morning stiffness [39]. McKellar et al. compared written information and cooking classes as means of Mediterranean diet implementation in women with RA living in socially deprived areas, showing a modest improvement in a number of measures of disease activity [40]. Finally, a recent randomized crossover trial did not obtain a significant reduction in DAS28 with a Mediterranean anti-inflammatory diet plus probiotics in RA patients [41]. Diet habit is also strictly interconnected with the gut microbiota. The human digestive system for example cannot digest several plant-derived complex carbohydrates present in cereals, vegetables, and fruits that are degraded and fermented by intestinal bacteria [11]. It is known that the western diet, rich in animal protein and saturated fats, can enhance the Bacteroides enterotype, whereas the Prevotella-driven enterotype is prevalent in carbohydrate and fiber-based diet [11]. The effect of the Mediterranean diet on microbiota composition has been already reported in healthy subjects. Mitsou EK et al. showed that a high adherence to the Mediterranean diet was characterized by lower *E. coli* counts and increased levels of *Candida albicans*; moreover, a positive correlation of MedDietScore with total bacteria, bifidobacteria:E. coli ratio and relative share of Bacteroides and *C. albicans* was reported [17]. Considering that several abnormalities have been described in the gut microbiota of RA patients and that dietary habits can influence gut dysbiosis, diet has been suggested as a possible additional strategy for the management of these patients. However, we found only one study, performed in 2005, without using sequence analysis techniques, that focused on the effect of the Mediterranean diet on microbiota composition in RA patients [42]. These authors reported that neither the Mediterranean diet nor fasting treatments affect the intestinal flora in patients with RA. More recently, we have found that a varied diet was inversely associated with *Pasteurellales, Paraprevotellaceae, Paraprevotella, Blautia, Blautia coccoides, and Bacteroides eggerthii* in RA patients; however, this was not the aim of that study, so diet habit was only generically assessed [9]. Profiling gut microbiota in RA patients with high and low/moderate adherence to the Mediterranean diet, in our current study, revealed considerable differences between the two groups. Though not affecting alpha-diversity, in agreement with previous findings [43,44], high adherence to the Mediterranean diet considerably impacted on microbiota composition. A striking result was the drop, observed in high versus low/moderate adherence group, of Euryarchaeota phylum, which we previously found to be directly correlated with DAS [9]. Again, among the phyla, the Mediterranean diet promoted detection of Cyanobacteria, which were absent in low/moderate adherence patients. As previously discussed, these bacteria can produce metabolic compounds endowed with anti-inflammatory and immunosuppressant properties, which could be beneficial for RA patients [9]. It is worth noting that at family level, there is the decreased abundance of Clostridiaceae in subjects following the Mediterranean diet, since this family has been found enriched and associated with the arthritis phenotype in RA patients [45]. Another intriguing observation is the significant under-representation of Lactobacillaceae (and its related genus Lactobacillus) and the inhibition of *P. copri* at species level, in the high adherence group, which can suggest a healthier gut microbiota composition. In fact, a significant increase in Lactobacillaceae in RA patients with respect to healthy controls has been previously reported, as well as in mice susceptible to developing collagen-induced arthritis [6,9,46]. *P. copri* has been shown to have a role in the autoimmune mechanism underlying RA onset. Indeed, it was found enriched in the gut of newly-diagnosed RA patients [8]; moreover, an increased immunity towards *P. copri* was demonstrated in subjects suffering from RA and a sequence homology between disease-specific autoantigens and antigens from *P. copri* was described [47]. Together with an enrichment of *P. copri* in RA patients, Scher et al. also observed a lower abundance of *Bacteroides fragilis* [8], which is reported to induce regulatory T cells, a subset of T lymphocytes suppressing autoimmunity [48]. In our current study, high adherence to the Mediterranean diet produced a marked increase in *B. fragilis* abundance, which we speculate may benefit RA patients by suppressing autoreactivity and inflammation. To the best of our knowledge, this is the first study investigating the protective effect of adherence to the Mediterranean diet on disease activity in RA patients and its associations with the composition of the gut microbiota by using next-generation sequencing. The main limit of this study is the absence of a longitudinal analysis that does not permit inferring a certain cause–effect relationship between the Mediterranean diet, RA clinical outcome and gut dysbiosis. In conclusion, our findings suggest the protective role of the Mediterranean Diet on disease activity of RA patients supported by a healthy impact on microbiota composition. If confirmed by prospective studies on a larger population, these data underline the feasibility of adopting the Mediterranean diet as a supportive care to modulate the gut microbiota and improve disease activity on RA patients.

## Figures and Tables

**Table 1:** Main demographic, anthropometric, and clinical characteristics.

Age, years	60.50 (53.00–69.5)
F (%)	88 (33)
BMI	24.21 (21.15–26.15)
Smoke (%)	18 (33)
Disease duration, months	13.00 (8.50–17.50)
DAS28	3.70 (2.98–4.80)
RF positive (%)	65.00
ACPA positive (%)	63.33
CRP (mg/dl)	3.60 (2.00–8.75)
ESR (mm/hg)	20.00 (11.50–33.00)

Data are expressed as median (25–75th percentile) or percentages (%). DAS28 = disease activity score on 28 joints; BMI = body mass index; RF = rheumatoid factor; ACPA = anti cyclic-citrullinated peptide antibodies; CRP = C-reactive protein; ESR = erythrocyte sedimentation rate.

**Table 2:** Main demographic, anthropometric, and clinical characteristics of the patients, according to the adherence to Mediterranean diet.

	Low/Moderate Adherence (*n* = 40)	High Adherence (*n* = 20)	*p* Value
Age, years	59.50 (52.00–65.50)	66.00 (56.50–70.00)	ns
F (%)	30.00 (75.00)	20.00 (100.00)	0.023
BMI	24.45 (21.10–27.45)	23.40 (21.20–26.02)	ns
Smoke (%)	8.00 (20.00)	3.00 (15.00)	ns
Disease duration, months	11.50 (6.00–16.50)	15.00 (12.50–19.50)	0.033
DAS28	3.95 (3.10–5.06)	3.30 (2.87–3.75)	0.034
RF positive (%)	27.00 (67.50)	12.00 (60.00)	ns
ACPA positive (%)	27.00 (67.50)	11.00 (55.00)	ns
CRP (mg/l)	4.86 (2.35–10.15)	2.47 (1.00–5.50)	0.037
ESR (mm/hg)	20.00 (10.50–34.50)	20.00 (13.50–21)	ns

Data are expressed as median (25–75th percentile) or percentages (%). DAS28 = disease activity score on 28 joints; BMI = body mass index; RF = rheumatoid factor; ACPA = anti cyclic-citrullinated peptide antibodies; CRP = C-reactive protein; ESR = erythrocyte sedimentation rate.

**Table 3:** Association between the class of adherence to the Mediterranean diet and the main demographic, anthropometric and clinical parameters of the RA patients.

	Univariate		Multivariate	
	B Coeff.	*p*-Value	B Coeff.	*p*-Value
F (%)	−1.400	ns	-	-
Disease duration, months	−0.002	ns	-	-
CRP (mg/dL)	0.0250	ns	-	-
Class of adherence to Mediterranean diet	−1.021	0.047	−1.130	0.042
Therapy	0.055	ns	-	-
Age, years	0.033	ns	-	-
BMI	−0.008	0.042	−0.060	ns
Smoke (%)	−0.164	0.049	−0.264	ns
RF positive (%)	0.66	ns	-	-
ESR (mm/hg)	0.040	0.0165	0.040	0.017

DAS28 = disease activity score on 28 joints; BMI = body mass index; RF = rheumatoid factor; anti-CCP = anti cyclic-citrullinated peptide antibodies; CRP = C-reactive protein; ESR = erythrocyte sedimentation rate.
